# An Audit of the Completion of the Admission Clerking Form and Physical Examination for Patients Admitted to the General Adult Inpatient Wards in Mersey Care NHS Foundation Trust

**DOI:** 10.1192/bjo.2025.10604

**Published:** 2025-06-20

**Authors:** Vivian Mgbachi, Roweida Sammour, Declan Hyland

**Affiliations:** Mersey Care NHS Foundation Trust, Liverpool, United Kingdom

## Abstract

**Aims:** Individuals with a severe and enduring mental illness (SMI) are at increased risk of poor physical health and demonstrate a higher premature mortality rate and reduced life expectancy of 15 to 20 years, compared with the general population. Despite being at increased risk of physical health comorbidities, individuals with an SMI are less likely to engage with physical health assessment and less likely to engage with required monitoring of any physical health comorbidity in the primary care setting. Admission to a psychiatric ward provides an opportunity to “screen and intervene”. Mersey Care NHS Foundation Trust has a policy for physical health assessment and monitoring for patients following admission to the ward. This audit aimed to assess level of compliance with the policy on the Trust’s general adult inpatient wards.

**Methods:** The patient list for nine of the general adult inpatient wards in the Trust was obtained, forming a sample of 160 inpatients. The electronic patient record for each inpatient was reviewed to determine whether an admission clerking form had been completed within 24 hours of admission, as recommended in the Trust’s policy, and whether a physical examination had been completed within 24 hours of admission, as recommended in the Trust’s policy. The level of completeness of the physical examination was also reviewed.

**Results:** 124 (78%) of the 160 inpatients had had an admission clerking completed within 24 hours of admission to the ward. Only 83 (52%) inpatients had a physical examination completed within 24 hours of admission to the ward. Of the 83 inpatients, 71 (86%) had a cardiovascular examination completed, 75 (90%) had a respiratory examination completed, 69 (83%) had a gastrointestinal examination completed, and only 63 (76%) inpatients had a neurological examination completed.

**Conclusion:** This audit highlighted that current practice indicates need for improvement, both from a patient safety and quality of patient care perspective. There is a need for increased emphasis on ensuring that any new admission is clerked in, and a physical examination is completed to determine that the patient is medically fit enough to remain on the ward and whether any physical health interventions are required on admission. The physical examination should be a complete one, particularly with psychotropic medication having the potential to cause neurological side effects. There should be a daily review of any patients who decline a physical examination on admission, with attempts made to engage them and complete this task as promptly as possible.

